# Integrating gender analysis into research: reflections from the Gender-Net Plus workshop

**DOI:** 10.1016/j.eclinm.2024.102728

**Published:** 2024-07-13

**Authors:** Christopher R. Cederroth, Brian D. Earp, Hernando C. Gómez Prada, Carlotta M. Jarach, Shlomit A. Lir, Colleen M. Norris, Louise Pilote, Valeria Raparelli, Paula Rochon, Nina Sahraoui, Cassandra Simmon, Bilkis Vissandjee, Chloé Mour, Mathieu Arbogast, José María Armengol, Robin Mason

**Affiliations:** a Department of Physiology and Pharmacology, Karolinska Institutet, Stockholm, Sweden; b Translational Hearing Research, Tübingen Hearing Research Center, Department of Otolaryngology, Head and Neck Surgery, University of Tübingen, Tübingen, Germany; c Uehiro Centre for Practical Ethics, University of Oxford, Oxford, England; d Yale-Hastings Program in Ethics and Health Policy, Yale University, New Haven, CT, USA; e University of Murcia, Murcia, Spain; f Laboratory of Lifestyle Research, Department of Medical Epidemiology, Istituto di Ricerche Farmacologiche Mario Negri IRCCS, Milan, Italy; g The Louis and Gabi Weisfeld School of Social Work, Bar Ilan University, Israel; h Faculties of Nursing, Medicine and School of Public Health, University of Alberta, Edmonton, Alberta, Canada; i McGill University Health Center Research Institute, Montreal, Quebec, Canada; j Department of Translational Medicine, University Center for Studies on Gender Medicine, University of Ferrara, Ferrara, Italy; k Women's Age Lab, Women's College Hospital, Department of Medicine and RTO/ERO Chair in Geriatric Medicine, University of Toronto, Canada; l Centre for Sociological and Political Research in Paris, CRESPPA, CNRS, Paris, France; m European Centre for Social Welfare Policy and Research, Vienna, Austria; n Faculté des Sciences Infirmières, Centre de Recherche en Santé Publique, Université de Montréal, Centre de Recherche SHERPA, Montréal, Québec, Canada; o Mission pour la Place des femmes au CNRS, France; p Mission pour la Place des femmes au CNRS, CEMS, Cresppa-GTM, France; q Departamento de Filología Moderna, Universidad de Castilla-La Mancha, Spain; r Women's College Research Institute, Women's College Hospital and Dalla Lana School of Public Health, University of Toronto, Canada

**Keywords:** Gender, Sex, Intersectional, Health research, Social sciences, Humanities

## Abstract

Gender equality has been a crosscutting issue in Horizon 2020 with three objectives: gender balance in decision-making, gender balance and equal opportunities in project teams at all levels, and inclusion of the gender dimension in research and innovation content. Between 2017 and 2022, the EU funded, in collaboration with national agencies, 13 transnational projects under “GENDER-NET Plus” that explored how to best integrate both sex and gender into studies ranging from social sciences, humanities, and health research. As the projects neared completion, forty researchers from these interdisciplinary teams met in November 2022 to share experiences, discuss challenges, and consider the best ways forward to incorporate sex and gender in research. Here, we summarize the reflections from this workshop and provide some recommendations for i) how to plan the studies (e.g., how to define sex and/or gender and their dimensions, rationale for the hypotheses, identification of data that can best answer the research question), ii) how to conduct them (e.g., adjust definitions and dimensions, perform pilot studies to ensure proper use of terminology and revise until consensus is achieved), and iii) how to analyze and report the findings being mindful of any real-world impact.

## Introduction

Over the last two decades, there has been growing recognition among research funding agencies, publishers of scientific and medical journals, and policy decision-makers that sex (a biological variable) and gender (a sociocultural variable) influence research and research outcomes. Consequently, it has become increasingly important to integrate considerations of sex and gender into all branches of research to boost accuracy, provide high-quality science, improve reproducibility and generate innovative, equitable and inclusive findings.^1^ As a result, the US National Institutes of Health (NIH), the Canadian Institutes of Health Research (CIHR), and the European Commission (EC) began outlining expectations that sex and gender be integrated in funding submissions^s^ and provided some guidance on how to do so (e.g., CIHR Institute of Gender and Health training modules; Gender-Net IGAR).^t^^,^^u^ Along with the different terms used to capture this approach in research (e.g., sex and gender dimensionality; sex, gender and diversity analysis (SG&DA),^2^ or in policies (e.g., sex and gender analysis plus (SGBA+); equity diversity and inclusion initiatives (EDI)],^3^ there are also differences in recommended practices for integrating these factors into studies and the extent to which they should be applied.^4^, ^5^, ^6^ Although many national funding institutions are lagging in enacting similar requirements,^2^^,^^7^ some peer reviewed journals have begun requiring the inclusion of sex and/or gender-specific details in manuscript submissions.^8^^,^^9^ Yet, the effective integration of gender, as distinct from sex but often interacting with it, remains challenging with inconsistent uptake, practices, and results.^10^^,^^11^ In short, determining the best practices for incorporating sex and gender into diverse investigations and across varied study designs, remains a pressing issue.

### GENDER-NET plus: addressing societal and healthcare needs

In 2018, GENDER-NET Plus funded 13 transnational research projects from 12 countries and 382 researchers with the aim of advancing the gender dimension in health, social sciences, and humanities research (Table 1). Ten projects focused on health issues while the remaining three examined climate change; technologies and entrepreneurship; and, modern cinema and literature. Over the following five years, each of the funded projects explored how to best integrate both sex and gender into their studies. As the projects neared completion, the project leads (PIs) along with co-Investigators and Early Career Researchers from these interdisciplinary teams were invited to meet in November 2022 to share experiences, discuss challenges, and consider the best ways forward to incorporate sex and gender in research studies.

**Table 1:** Brief descriptions of the GENDER-NET Co-Plus funded project.

Acronym	Title	Topics	Key references
FUTUREGEN	Evolving gender differences in health & care across cohorts	• Sex, Gender and Health/Aging	12, 13, 14
GBV-MIG	Violence against women migrants and refugees: Analyzing causes and effective policy response	• Gender-Based Violence • Sex, Gender and Health	^15^
G-DEFINER	Gender difference in side effects of immunotherapy: a possible clue to optimize cancer treatment	• Sex, gender and health	^16^
GENDER-ARP	Addiction, health risks and recovery in context of social precarity: how to better address complex needs taking into account gender and life stages	• Sex, Gender and Aging • Sex, Gender and Health	^17^^,^^18^
GENPATH	A life course perspective on the GENdered PATHways of social exclusion in later life, and its consequences for health and well-being	• Sex, Gender and Aging • Sex, Gender and Health	^19^^,^^20^
GENRE	Overcoming the entrepreneurial ecosystem gender divide: a cross-cultural perspective	• Gender In Entrepreneurship and in the Innovation System	^21^^,^^22^
GOING-FWD	Gender Outcomes INternational Group: to Further Well-being Development	• Sex, Gender and Health	^23^^,^^24^
IKASCADE	Identifying Key prescribing CASCADes in the Elderly: a transnational initiative on drug safety	• Sex, Gender and Aging	^25^
MASCAGE	Gendering age: representations of masculinities and ageing in contemporary European literatures and cinemas	• Sex, Gender and Aging	^26^
POSITIVMASC	Masculinities and violence against women among young people- Identifying discourses and developing strategies for change using a mixed method approach	• Gender-Based Violence	^27^^,^^28^
RHCFORFGC	Sharing actions and strategies for Respectful and equitable Health Care for women with FGC/M.	• Sex, Gender and Health	^29^^,^^30^
SEQUAL	Social-ecological relations and gender equality: dynamics and processes for transformational change across scales	• Gender Dimension in Climate Behaviour and Decision-Making	^31^
TIGER	The combined role of genetic and environmental risk factors in the gender-specific development of severe tinnitus	• Sex, Gender and Health	^32^^,^^33^

Forty researchers gathered for a two-day workshop that included scientific reports from the funded project teams in the form of presentations, small group discussions, and plenary sessions. Discussion topics included: i) integrating patients or community members most affected by or concerned with the issue into the research process; ii) using administrative data that is only disaggregated into male/female binaries; and iii) ensuring those participating on proposal evaluation panels have sufficient training to effectively evaluate the sex and gender dimensions of a proposal. The plenary sessions focused on the broader funding climate and the need to explore ways of increasing the sharing of knowledge and learning from different disciplines. The various countries, cultures, projects, and participants’ professions and disciplines ensured lively discussions as all wrestled with drawing useful conclusions from across the studies. Here, we share some elements of our reflections to stimulate research on sex and gender as well provide recommendations on what should be considered at each step of the study: planning, conduct/process, analysing and reporting (Panel 1).

*Panel 1* **Recommendations from the workshop: clear definitions and justification in all stages of research**

1) Planning
  a) Define sex and/or gender (and specify the relevant dimensions) as they will preliminarily be understood for purposes of the research
  b) Provide a clear rationale with hypotheses about the role of sex and/or gender so defined, with adequate operationalizations
  c) Qualify or adjust definitions/language based on factors involving participants
  d) Identify data that can best answer the questions: prospective or retrospective
  e) Construct a multidisciplinary (and relevantly diverse) team
2) Conduct/Process
  a) Adjust definitions, dimensions, operationalizations during a pilot phase based on relevant details from country/culture/context
  b) Pilot your terms to ensure understanding, accuracy, and relevance
  c) Build a terminology chart
  d) Be open to going back and forth with revisions
3) Analyzing and Reporting
  a) Clarity of terms must be consistent throughout and in keeping with the process
  b) Emphasize why the terms (and definitions, operationalizations, etc.) have been chosen and how they bear on the research question or findings, being mindful of any real-world impact

### Definitions of sex and gender

With teams from a variety of backgrounds, it was important to explore definitions of sex and gender in our discussions. What clearly emerged from the study presentations and ensuing discussions, was that *‘sex’* and *‘gender’* often interacted and were difficult to tease apart. Defining and documenting gender was found to be complex, challenging, and was approached in diverse ways in the funded studies. When the research question focused on biological features, then *‘sex’* rather than *‘gender’* appeared be the more appropriate term. In such cases, specific and relevant aspect(s) of sex (e.g., sex assigned at birth, sex chromosome, sex hormones, sex organs) could be used to describe the population, although it was noted that care should be taken to avoid suggesting that ‘sex’ is a simple binary; doing so misrepresents the diversity of humans. Conversely, when aspects of a person's psychology (e.g., self-concept) or social positioning (e.g., differential treatment based on their perceived or actual reproductive features) were found most relevant, then the term *‘gender’* was deemed more appropriate. We noted that in social sciences research, sociocultural analysis needed to also include important factors such as class, race, poverty level, ethnic group and age (EIGE). Definitions of *‘gender’* in these studies emphasized that unequal power relations between dominant (e.g., white cisgendered) and underserved (e.g., older, substance using, migrant) groups and individuals, required thoughtful methodological choices.

### The need to consider gender beyond the binary

During the workshop, it was agreed that, while several initiatives have been taken to improve study design descriptions and reporting in the scientific literature,^34^, ^35^, ^36^ existing databases and resources often describe sex as either male or female, and gender as either woman or man—oversimplifications which are no longer appropriate. Several practical challenges were noted during the workshop in this regard and there were differing views about how best to integrate appropriate considerations of sex and/or gender in research. Though tempting, creating a single ‘centralized’ authoritative voice (e.g., guidelines) were thought to run the risk of being dogmatic/political and contrary to scientific freedom and debate. As we struggled with this issue, it was noted that doing a gender/sex-based analysis poorly, or in a ‘pro forma’ manner (to ‘jump through a hoop’), may often be worse than not conducting the analysis at all. Consequently, clarity in what constitutes *‘gender’* in any one study is necessary; only then can the adequacy of the conceptual distinctions drawn, the operationalization and relevance to the research question, be duly evaluated. In the context of research, the extent to which, and in what way(s) grouping humans by *‘sex’* and/or *‘gender’* is appropriate, must be continuously evaluated in relation to the research question at each stage of the scientific process: from the study design, the initial grant application (where relevant), ethics license, creation of materials, sampling procedure, statistical analysis plan (ideally pre-registered), to the actual analysis and write-up of the results.

As a result, we suggest there cannot be a standardized measure of *‘gender’;* the items and instruments to be used need to be evaluated and determined depending on the study objectives. The GOINGFWD project proposed several methods to guide the selection of gender-related variables, ranging from gender identity, to gender roles and gender relations.^23^ However, in addition to asking participants about their *‘sex’* at birth with options *‘male’, ‘female’,* or *‘intersex’*, the minimum options for gender should be *‘woman’, ‘man’, ‘non-binary’* and *‘something else’*. When appropriate, personality traits that may also impact the outcome of the study can be assessed using personality questionnaires such as Bem Sex-Role Inventory,^37^, ^38^, ^39^, ^40^ Big-5,^41^ or Myers-Briggs.^42^

### Challenges in retrospective and prospective studies

It was observed that in Sweden, the same word “*kön*” is used to define either sex or gender in a binary way (i.e., *man, kvinna*). It was thus difficult to infer whether the researchers or the study participants were described by their sex or gender. When using population registries, the researchers relied on the information available on the sex of a person from the population register and visible in the penultimate digit of the social security number. However, whether this is the identity that was given at birth based on the phenotypic sex, or whether the legal sex has changed over time due to a shift in gender identity, is unclear.

Challenges were also found when studies involved secondary analyses of existing quantitative datasets, where–despite having data disaggregated by sex–gender had not been collected and could only be inferred from other available variables such as socio-economic status, employment, or race (variables not always collected or in some jurisdictions not permitted to be collected). The GOINGFWD project needed to create a “harmonized dataset” that included variables from across all the partners’ datasets.^23^

In health research, the study from Nielsen et al. proved very informative in measuring Gender as a Sociocultural Variable (GASV), as a complement to Sex as a Biological Variable (SABV).^43^ The study identified from 44 survey items and 11 original constructs, 7 gender-related variables (i.e., caregiver strain, work strain, independence, risk-taking, emotional intelligence, social support, and discrimination) that were relevant to capturing gender dimensions in relation to health. This Stanford Gender-Related Variables for Health Research questionnaire was made available in the supplemental materials of the publication.^43^

### Sex, gender and the dimension(s) of intersectionality

In specific projects such as GBV-MIG and POSITIVMASC, integrating sex and gender were not sufficient to capture the particular experiences under investigation. In these studies, additional identity-related factors needed to be considered such as migrant and immigrant women's experiences of gendered violence. To complement the focus on gender, the researchers drew on intersectionality theory. Intersectionality (a term attributed to the Combahee River Collective, later formalized as a theory in 1989 by Black feminist and critical legal scholar, Kimberlé Williams Crenshaw)^44^ describes the ways that social categories such as race, class, and gender, as they apply to a given individual or group, are politically contextualized or situated, creating axes of relative oppression or privilege. The way in which individuals are positioned by intersecting categories (e.g., gender, race, class) shapes their life experiences and well-being. Examining these intersecting variables required elaborate planning as they cannot be properly considered as additive or cumulative and consideration of both micro (e.g., identities, individual structure) and macro (e.g., institutions, policy and legal aspects, social structure) level factors were needed in developing the research questions and conducting the studies.

### The importance of “how” to collect the data

During the workshop discussions, it became clear that it was critically important to consider not only what data to collect but also the ways in which data are collected. When considering gender, gender-related factors, and intersectionality, qualitative research designs offer more possibilities for sensitive data collection that may serve either as a pilot study to inform quantitative study design, and/or as further exploration of issues that emerge from a survey (qualitative post-surveys). Selecting factors likely to be relevant to your research may become clear during the literature review phase of proposal development or emerge during the study itself. It may be helpful to remain open to adding other methods (mixed methods) to the original study design in post-hoc analysis. As data collection proceeds, it is important to review who is being included in the research and who may be excluded. More participatory strategies that involve those most concerned with the issue may help ensure that privileged, less privileged, and underserved groups are represented; appropriate terms and language are used; no factor is prioritized over another (e.g., ethnicity over class); questionnaires cover the essential items; and that the political dimensions (the consequences of the research) are not ignored. The intersection of gender with other aspects of identity should be considered when developing data collection strategies as well as during data analysis and publication of study results.

The team's diversity and multidisciplinarity can help ensure that a range of perspectives are included in study plans and processes, leading to improved science.^45^ Researchers should ensure that they have considered all relevant points and perspectives before designing and carrying out a study. Why is this issue seen as scientifically interesting (and to whom)? What is the research question and what does it leave out? How will this study be perceived by the community? What are the covert and overt gendered aspects of the research? Can aspects of intersectionality be discerned and noted for a greater understanding of their effect on the examined phenomenon? Could the research inadvertently harm the population or specific communities, groups, or individuals? As researchers and knowledge producers, we are in a position of privilege and power, and with it, afforded a certain level of trust. One must consider how the findings that result from one's research may be interpreted and used, particularly when the study concerns vulnerable individuals or groups and sensitive issues. This does not mean that the possible misuse of valid research results should necessarily prevent the research from going forward; however, all due efforts to mitigate such misuse or associated harmful outcomes (e.g., by heading off foreseeable misunderstandings) should be made. Similarly, while maintaining a high standard for accuracy, clarity, and predictive potential in drawing conceptual distinctions, the definitions and words being used to describe a population can increase, or help to minimize, any stigma and challenges they may face. It is also recommended to use gender-neutral language when referring to groups or persons of unknown or indeterminate gender and to acknowledge the diverse implications for various groups within the research through choice of language. These are important points to consider during the design and framing of a study and can determine how it will be perceived and received by different audiences.

Most importantly, adopting a gendered approach should include a focus on the research process. One needs to reflect on how the research is done and not only about what. In this regard, policies such as those promoting Equity, Diversity and Inclusion (EDI) allow one to reflect on the composition of a workforce (“*who”* is at the office desk, on the team, or in the laboratory) and increase the participation of women and racial minorities.^3^ We believe that such policies supports the adoption of a gender-sensitive approach to the various steps of the research processes and increases the diversity of the research community with the goal of achieving equity. This entails reflecting for instance on:

(1) ***Research and human resources management*** – It is important to reflect on potential gendered inequalities, especially within project-based research: Is the research group diverse and gender inclusive? Are maternity and parental leave rights respected? Is it possible to prolong the project if some members take parental leave? Is the work environment respectful of these rights? Gender-responsiveness starts within our institutions; ensuring a gender-responsive budget and time management procedures that avoid reproducing the gendered inequalities that permeate the labor market, are important obligations of gender-aware researchers.
(2) ***Data collection procedures*** – The question of who is included in a study, either as an interview participant or community member of the research team, requires consideration of the material and financial provisions for the research project: for instance, will interviewees, community members, or other stakeholders on the research team be remunerated for their time? Will participants' expenses related to childcare or transportation for example, be covered?
(3) ***The potential impacts of the research*** – An intersectional and gendered approach to research is inherently tied to a commitment to social justice, equity, and gender equality. Responsible research should include consideration of the societal impact of the research; this may mean adjusting the planned research implementation and knowledge translation activities. There are ethical implications to much of what we study and the knowledge that is produced. It can be helpful to engage those most affected by the study to help with appropriate dissemination and knowledge translation.

In 2019, the European Gender Equality Commission was established to help ensure the mainstreaming of gender equality into all Council of Europe policies. The Task Force on Equality outlined mechanisms, policies, and actions to challenge structural discrimination and stereotypes. In March 2020, the European Union adopted its directives, which outlined actions needed to advance gender equality in Europe and beyond. Due to the complexity associated with sex and gender, there is a clear need for interdisciplinary research to generate a holistic understanding of social inequities that would lead to better recommendations, strategic interventions and programs, and more effective policies to reduce them.

## Outstanding questions

Ensuring that education about the “*whys*” and “*hows*” of intersectional sex and gender integration needs to be promoted across the entire range of research endeavours. For instance, to enable the continued growth of sex and gender in research, even null findings need to be reported to alert future researchers know where to begin their investigations. As integrating sex and gender analysis in research becomes more frequent, the urge to share experiences and build frameworks grows stronger. By sharing our experiences, methods, and tools with the scientific community, we hope to advance consideration of intersectional sex and gender integration in all research.

## Contributors

C.R.C., B.D.E., H.D.G.P., C.M.J., S.A.L., C.M.N., L.P., V.R., P.R., N.S., C.S., B.V., C.M., M.A., J.M.A., R.M., contributed equally to the manuscript (conceptualization, funding acquisition, writing original draft, and final editing) and approved the final version.

## Declaration of interests

All authors declare no competing interests.

## Footnotes

s https://research-and-innovation.ec.europa.eu/strategy/strategy-2020-2024/democracy-and-rights/gender-equality-research-and-innovation_en#gender-equality-in-horizon-europe.

t https://igar-tool.gender-net.eu/en.html.

u https://eige.europa.eu/sites/default/files/d3.11_manuals_with_guidelines_on_the_integration_of_sex_and_gender_analysis_into_research.pdf.
